# Unexpected resilience in relict *Abies pinsapo* Boiss forests to dieback and mortality induced by climate change

**DOI:** 10.3389/fpls.2022.991720

**Published:** 2022-12-23

**Authors:** Álvaro Cortés-Molino, Juan Carlos Linares, Benjamín Viñegla, Víctor Lechuga, A. Enrique Salvo-Tierra, Antonio Flores-Moya, Ismael Fernández-Luque, Jose A. Carreira

**Affiliations:** ^1^ Centro de Estudios Avanzados en Ciencias de la Tierra, Energía y Medio Ambiente (CEACTEMA), Universidad de Jaén, Jaén, Spain; ^2^ Departamento de Botánica y Fisiología Vegetal, Universidad de Málaga, Málaga, Spain; ^3^ Departamento de Sistemas Físicos, Químicos y Naturales, Universidad de Pablo Olavide, Sevilla, Spain

**Keywords:** climate change, forest resilience, ecosystem dynamics, remote sensing, multitemporal analysis

## Abstract

Acute and early symptoms of forest dieback linked to climate warming and drought episodes have been reported for relict *Abies pinsapo* Boiss. fir forests from Southern Spain, particularly at their lower ecotone. Satellite, orthoimages, and field data were used to assess forest decline, tree mortality, and gap formation and recolonization in the lower half of the altitudinal range of *A. pinsapo* forests (850-1550 m) for the last 36 years (1985-2020). Field surveys were carried out in 2003 and in 2020 to characterize changes in stand canopy structure and mortality rates across the altitudinal range. Time series of the Normalized Difference Vegetation Index (NDVI) at the end of the dry season (derived from Landsat 5 and 7 imagery) were used for a Dynamic Factor Analysis to detect common trends across altitudinal bands and topographic solar incidence gradients (SI). Historical canopy cover changes were analyzed through aerial orthoimages classification. Here we show that extensive decline and mortality contrast to the almost steady alive basal area for 17 years, as well as the rising photosynthetic activity derived from NDVI since the mid-2000s and an increase in the forest canopy cover in the late years at mid and high altitudes. We hypothesized that these results suggest an unexpected resilience in *A. pinsapo* forests to climate change-induced dieback, that might be promoted by compensation mechanisms such as (i) recruitment of new *A. pinsapo* individuals; (ii) facilitative effects on such recruitment mediated by revegetation with other species; and (iii) a ‘release effect’ in which surviving trees can thrive with fewer resource competition. Future research is needed to understand these compensation mechanisms and their scope in future climate change scenarios.

## 1 Introduction

Extreme climate events such as severe droughts and heat waves are expected to increase due to climate change (Jentsch & Beierkuhnlein, 2008; Swain et al., 2020), thus challenging the adaptive capacity of forests worldwide to these new scenarios (Lindner et al., 2010). Disturbances are an essential component of forest ecosystem dynamics (Attiwill, 1994) that plays an important role in tree recruitment and turnover, and in stand canopy structure and compositional changes (Seidl et al., 2011; Kuuluvainen et al., 2021). Minor disturbances at the local scale promote forest gap opening and subsequent closing processes which, due to spatio-temporal asynchronies, results in landscape diversification and shifting-mosaic and patch dynamics at the landscape meta-scale (Picket & White, 1985). However, the increasing occurrence and severity of climate extreme events might threaten these natural disturbance dynamics and compromise both forest resistance and resilience capabilities (Gazol et al., 2018a).

Resistance has been defined as the ability of ecological systems to persist or minimize damage through a disturbance event (Connell & Ghedini, 2015; Sánchez-Pinillos et al., 2019). Once disturbance drivers are alleviated, the ecosystem’s capacity and speed of recovery, or resilience (Gunderson, 2000), has been assessed through three approaches: from an engineering point of view, which considers a single stability state; from an ecological perspective based on the existence of multiple stabilities; and under a social-ecological criterion which assumes the maintenance of the current state (Nikinmaa et al., 2020). Resistance and resilience are key concepts to understanding ubiquitous forest dieback processes currently observed at the global scale (Allen et al., 2010), and to assess an increasing vulnerability to tree mortality and forest die-off from hotter drought in the Anthropocene (Allen et al., 2015). Climate-driven forest decline and its consequences in terms of reduced tree-growth, increased mortality, and forecasted distributional shifts at lower ecotones have been intensively studied in the last decades (e.g., Linares et al., 2009a; Camarero et al., 2015; Restaino et al., 2019). However, much less attention has been paid to the resilience component following drought-induced dieback events, including recovery processes at the individual (tree-growth responses; Gazol et al., 2018b; DeSoto et al., 2020) and community (forest vegetation recovery; Lloret et al., 2004, Lloret et al., 2012) levels. This is surprising since it is well known that the post-mortality phase following other types of disturbances, such as wildfires and land-use change, is key to understanding the mid-to long-term consequences in terms of species abundance changes and compositional shifts (Guo et al., 2018; Yu et al., 2019; Coop et al., 2020).

Post-mortality dynamics may just result in minor changes in the spectrum of functional traits at the community level, but with the persistence of the main structure of the system; that is, the previously dominant tree species maintains its dominant/co-dominant status through the growth of survivor adults and regeneration (Suarez & Lloret, 2018). Or it may lead to local extinctions of more drought-vulnerable species, replacement by drought-resistant ones, and thus to state shifts if the forest ecosystem is forced beyond its resistance and resilience limits (Anderegg et al., 2013). Under recurrent extreme climate events, the whole landscape structure can change, even turning forests into shrublands, as has been reported, among others, for Mediterranean cork oak (*Quercus suber* L.) forests turning to *Cistus ladanifer* L. shrub formations under persistent drought scenarios and recurrent wildfires in South Portugal (Acácio et al., 2009). All these consequences may be especially detrimental for relict and endangered tree species, whose current distribution areas usually hold environmental conditions near their tolerance limits (Hampe & Jump, 2011).

Assessing post-mortality forest recovery and compositional shifts is hampered by the fact that mortality events tend to be patchy and regeneration trajectories heterogeneous across a range of spatio-temporal scales (Breshears et al., 2009). Thus, to achieve a comprehensive characterization of post-drought forest resilience, a combination of methodological approaches, including both field plot-based and remotely sensed data, is needed (Dorman et al., 2015; Gazol et al., 2018b). The spatial resolution of available long-term satellite data (e.g., 30m pixel size for Landsat data) is coarser than needed to account for detailed canopy structural changes (e.g., gap dynamics, canopy height) and demographics processes (e.g., regeneration) at the stand level. Thus, multi-temporal aerial photographs combined with sequential field sampling are also used to gather both spatially extensive information and detailed plot-based data which together inform on changes in gap dynamics, mortality rates, tree status, forest density, and basal area (Vilà-Cabrera et al., 2012; Baguskas et al., 2014). These complementary measures can help answer if drought-induced mortality and subsequent tree growth and regeneration of the dominant tree species, and gap colonization by other species, scale up or not too persistent changes in vegetation composition and productivity.

*Abies pinsapo* Boiss. is a climate-relict fir species, endemic to the SW of the Iberian Peninsula, which is currently subjected to a Mediterranean-type climate seasonality. Its relict and endemic nature together with its climatic sensitivity render *A. pinsapo* the most vulnerable tree species of the Iberian Peninsula (Arista et al., 2011), and as one of the most vulnerable fir species among the group of Circum-Mediterranean firs, to drought-induced growth decline and mortality (Sánchez-Salguero et al., 2015). As early as the beginning of the 1990s, severe decline and extensive dieback symptoms were reported in the largest of the remaining *A. pinsapo* populations (Yunquera Forest, Sierra de las Nieves National Park) (Linares et al., 2009a), which have been associated to recurrent droughts and long-term warming trends (Linares et al., 2011). Recurrent dieback events, observationally showing complex spatio-temporal dynamics, have taken place since then and continue today (Navarro-Cerrillo et al., 2022). Therefore, this forest provides a unique opportunity to study the resilience component of vulnerability throughout a three-decade-long process of dieback events and post-mortality dynamics affecting a highly drought-sensitive tree species.

This work focuses on the study case of *A. pinsapo* at the Yunquera forest and aims to assess its conservation status by (i) characterizing the spatio-temporal dynamics of forest productivity at the landscape level using Landsat NDVI time series, (ii) characterizing the spatio-temporal balance of canopy gaining/loss through aerial orthoimages, and (iii) assessing changes in *A. pinsapo* tree status and mortality at the forest stand level through two field sampling campaigns separated in time by almost two decades.

## 2 Materials and methods

### 2.1 Study site and field survey

The study site was placed in the Yunquera pinsapo forest, the largest remaining forest patch of the Spanish fir (*A. pinsapo*). We chose this location because (i) it hosts most of the current *A. pinsapo* populations; (ii) it was the first one where conservation measures were adopted because of public ownership of the forest; and (iii) acute episodes of decline and stand stagnation have been observed there since 1994 (Linares et al., 2009b; Navarro-Cerrillo et al., 2022).

At present, *A. pinsapo* total distribution area is approx. 4000 ha in north-facing slopesabove 900 m a.s.l. at coastal mountains of the Baetic Range, including somewhat continuous *A. pinsapo* forest patches (accounting for approx 2000 ha) as well as the presence of isolated trees and small stands. This tree species is included on the IUCN Red List of Threatened Species as ‘Endangered’ (Arista et al., 2011) and is declared as ‘At risk of extinction’ under regional government law (Boletín Oficial de la Junta de Andalucía, 2011).

The annual mean temperature is 14.7 °C and annual precipitation ranges from 800-1600 mm in the Yunquera forest. Rainfall patterns are distinctly Mediterranean, with approx. 80% of annual precipitation falling from October to May, followed by a long summer drought.

**Figure 1:**
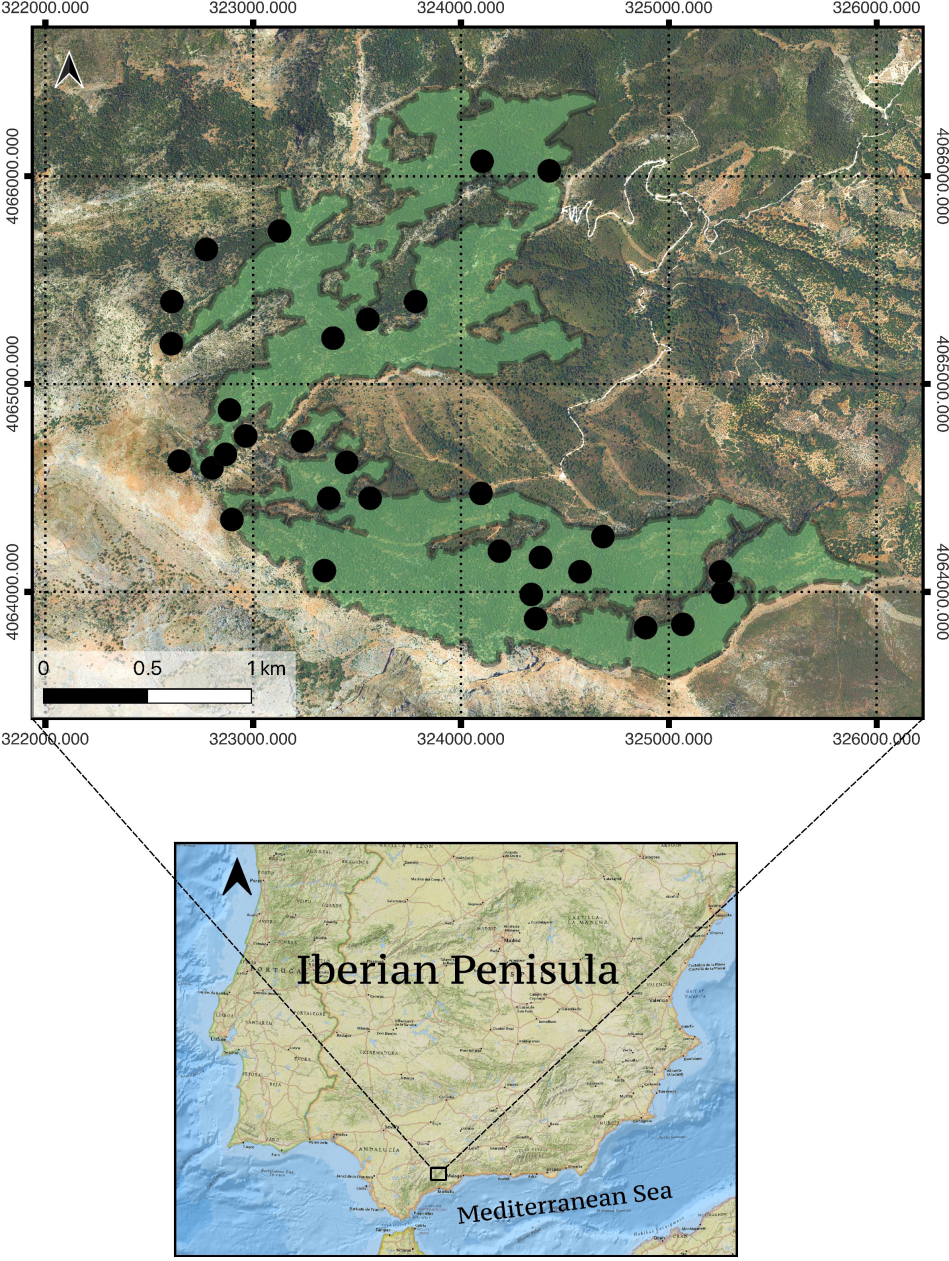
Study site location, Sierra de las Nieves National Park (Andalusia, south Spain). Green color patches show the areas of pure *Abies pinsapo* stands in the Yunquera forest (ca. 300 ha) used for both the Landsat and orthomosaic multitemporal analyses (resulting from the application of a -30m buffer to the pure *Abies pinsapo* mask to preclude the problem of mixed pixels; see 2.2 section). Black dots denote the location of the 31 plots used in the 2003 and 2020 field surveys. Since the ‘grain’ component of the study scale in the remote sensing approach -30mx30m for Landsat imagery- is coarser than that applied in the field-survey approach, some small pure stands included in the field-survey were excluded after applying the -30m buffer and are not accounted for in the Landsat imagery analysis. This is why a few black dots in the Figure are outside the green area. The coordinate sytem employed was EPSG:25830 - ETRS89/UTM zone 30N.

The study area was delimited as the masks within the Yunquera forest defined as ‘dominantly or pure and well-structured *A. pinsapo* forests’ (70-100% of *A. pinsapo* cover; Navarro Cerrillo et al., 2013) in the GIS of the Programme for the Recovery of *Abies pinsapo* (Andalusian regional government; https://www.juntadeandalucia.es/medioambiente/portal/). Areas with mixed stands (with *Quercus sp* and *Pinus halepensis Mill.*) and scattered *A. pinsapo* trees within the Yunquera forest were thus excluded from the study to avoid confusing results. Overall, the studied forest stands comprise over 300 ha and an 800-1550 m altitudinal gradient (**Figure 1**).

The field surveys were performed in 2003 and 2020. The plots were selected using an extensive, stratified random sampling, in equifrequent classes every 200 m in elevation. We sampled the same thirty-one *A. pinsapo* plots (150-m^2^) both in 2003 (Linares & Carreira, 2009) and 2020. Due to technical limitations in 2003 survey, we measured different trees for both surveys through random sampling within plots. We recorded tree diameter (considering only trees with diameter at breast height *dbh* > 3 cm), basal area, and recent mortality. In both surveys, environmental variables (elevation, aspect, soil type, topography, structure, overstorey, and understory types) and biotic variables (basal area of *A. pinsapo* stumps, living and dead trees) were recorded. Data on dead trees refer to those which had died recently, i.e., since the late 1990s according to dendrochronological estimates based on a previous work that correlated visual wood decay status with dendrochronological death date (Linares et al., 2010b). Declining and dead trees were classified according to dieback (defoliation, needles brownness) and bark/wood decay symptoms: Class 1 for >2/3 of the crown green and retaining needles, Class 2 for defoliation in 1/3 of the crown, Class 3 for severe defoliation and brown needles, Class 4 for a total loss of needles and thin branches, Class 5 for a total loss of medium branches and almost absent bark, and Class 6 for stumps (**Supplementary Figure 1**). Two ANOVAs were performed: one to detect BAI tree classes changes in both field surveys and another to study BAI per altitudinal bands. In this second one, tree classes were grouped by Alive, Dead, and Stumps.

### 2.2 Climatic and radiation data

To quantify climate-growth relationships, monthly mean temperature (T, units in °C; 0.25° resolution) and radiation (R, units in W·m^-2^) were downloaded from the EOBS database v23.1e for the period 1985–2020 (Haylock et al., 2008) using the Climate Explorer webpage (https://climexp.knmi.nl/). To quantify drought severity, we used the Standardized Evapotranspiration Precipitation Index (SPEI; Beguería et al., 2014). SPEI is a powerful tool for supporting drought tolerance monitoring since it is built on a climatic water balance (Vicente-Serrano et al., 2010). Xu et al. (2018) applied SPEI to study severe drought responses in southwestern USA forests, based on several canopy traits. This index is a multi-scalar index that quantifies drought intensity based on the difference between precipitation and atmospheric evaporative demand for different periods, with negative values indicating drier-than-average conditions, and positive figures for wetter-than-average conditions. SPEI data were also obtained from the Climate Explorer webpage and downloaded at 0.5° resolution. All the variables were averaged at the seasonal time scale: prior autumn (‘aup’; including September, October, and November of the previous year), winter (‘wi’; December of the previous year, January, and February), spring (‘sp’; March, April, and May) and summer (‘su’; June, July, and August). Autumn of the current year was not included as NDVI data were obtained for August-September (see below).

### 2.3 Dynamic factor analysis of landsat data series (1985-2020)

The Normalized Difference Vegetation Index (NDVI) has been used as a proxy of vegetation biomass and forest physiological performance (Wang & Tenhunen, 2004), that is sensitive to drought and changes in forest structure (Gazol et al., 2018b), and allows capturing post-disturbance recovery by time series analysis (Kennedy et al., 2010). The behavior of time series can be reported through the estimation of common trends, which are patterns in data that highlight relevant changes along the series.

We run Semiautomatic Classification Plugin (SCP) from QGIS software to obtain the satellite images, acquired from Landsat 5 and 7 for 36 years (1985-2020). We employed red (0.63 - 0.69 µm) and near-infrared (0.77 - 0.90 µm) spectra data with 30m spatial resolution. For each year, we obtained the first free cloud image available from the mid-August to mid-September period, since it corresponds to the end of the dry season, when biomass and NDVI are better correlated (González-Alonso et al., 2006; Vaglio Laurin et al., 2016). The data were radiometrically normalized, stacked, clipped with the *A. pinsapo* ‘pure and well-structured forests’ vector layer, and then the normalized difference vegetation index-NDVI was calculated. To minimize the edge effect in the satellite images caused by a 30 m pixel size, the layer mask was buffered -30m, and also excluded perimetral pixels, so we were theoretically able to ensure dense *A. pinsapo* forest pixels.

Altitude and solar incidence (monthly sunshine levels at each pixel corrected by slope, facing, and relief shadowing effects, expressed in hours; ‘SI’ hereafter; Guerrero et al., 2014) have been reported as the main factors conditioning *A. pinsapo* presence according to niche distribution models (Navarro Cerrillo et al., 2021). Thus, the pixels of the final satellite layers were further assigned to three altitudinal bands and three solar radiation incidence value ranges. For this, we first created a precise Digital Terrain Model (DTM) of the area using aerial LIDAR point cloud data with 0.5 points·m^2^ resolution, obtained in 2015 by the PNOA project of the Spanish National Geographic Institute (https://centrodedescargas.cnig.es/CentroDescargas/catalogo.do?Serie=LIDAR). FUSION software was employed for point cloud processing, following the guidelines of McGaughey & Carson (2003). We extracted bare ground point cloud to create the DTM, and Landsat pixels were clipped into three elevation bands: 880-1150 m, 1150-1350 m, and 1350-1550 m (hereafter denoted as the 1150 m, 1350 m, and 1550 m altitudinal bands). Then, we used SI data extracted from a raster layer generated by the Andalusian Digital Solar Incidence Model (REDIAM website of the Andalusian Regional Government; https://portalrediam.cica.es/geonetwork/srv/api/records/cb75e8c3eb1bcca6df28f57809d652736fad7572). We selected November data since correlation with *Abies pinsapo* distribution is the highest for this month, even to correctly predict the presence of a few isolated trees in small patches (i.e, shady gullies) within extensive areas with no *A. pinsapo* fir (personal communication; J.B. López-Quintanilla, *A. pinsapo* regional coordinator). SI data files were also ranged into three values intervals (0-46 h, 46-130 h, 130-175 h; hereafter denoted as the 46 h, 130 h, and 175 h SI ranges) to mask the elevation bands and create layers to finally clip the satellite data along the 36 years by the altitudinal bands and SI range.

A total of 324 Landsat images were processed; they were stacked, converted to data frames, and extracted the mean and standard deviation for every year of each vegetation index. The final output (NDVI time series for each pixel by SI and altitudinal values) was subjected to Dynamic Factor Analysis (DFA), both on raw and on standardized NDVI data, to find common temporal trends by running Brodgar software (Zuur et al., 2007). DFA is a robust procedure to find common trends in time series, and has been widely used in fisheries (Zuur et al., 2003; Ogle, 2018) and landscape monitoring (Campo-Bescós et al., 2013). Finally, to assess the effects of SI and altitude on NDVI time series we used repeated measures ANOVA with its subsequent Bonferroni pairwise comparison test.

### 2.4 Linear mixed effect models

We fitted linear mixed-effects models (LMEM, thereafter) using the *nlme* package in R software (R Development Core Team 2021) for *A. pinsapo*-dominated forests NDVI along three elevation bands: low elevation (880-1150 m a.s.l.), mid elevation (1150-1350 m a.s.l.) and high elevation (1350-1550 m a.s.l.). In order to test for heteroscedasticity, residuals were tested against observed values, predicted values, time calendar, elevation, and first-order autocorrelation. Climate variables such as seasonal data of temperature, radiation and SPEI (see section 2.2) were included as fixed factors, and each NDVI pixel from the elevation bands was included as a random factor. The covariance parameters were estimated using the restricted maximum likelihood method, which makes estimates of parameters by minimizing the likelihood of residuals from the fitting of the fixed effects portion of the model (see further details in Zuur et al., 2009). For both DFA and LMEM analyses, we used an information-theoretic approach for multi-model selection, based on the Akaike Information Criterion (AIC) corrected for small sample sizes (AICc). ΔAICc represents the difference between the lowest AICc observed (best fitting model) and those of each sequent model tested, being the model with ΔAICc = 0 the best model observed. However, all the models with ΔAICc < 2 have similar substantial support. Therefore, the models among them with less number of explanatory variables were finally selected, following the maximum parsimony criteria (Burnham & Anderson, 2002). Residuals pattern (correlation) were tested for observed values, model predictions, time (calendar year), elevation, and first-order autocorrelation (i.e., the correlation between model residuals for the year *i* and the observed NDVI in the year *i*-1). Temporal trends of the residuals were modeled by the Loess smoothing method using a polynomial weight function of degree 3.

### 2.5 Spatio-temporal dynamics of canopy loss and gap opening

We used historical orthoimages (obtained by aircraft through the PNOA project), masked with the *A. pinsapo* distribution vector layer, to study the spatio-temporal dynamics of *A. pinsapo* cover changes (gap opening and recovery) along the study area. Due to limitations in the number of years of data capture and varying image qualities among the available years, the final set of images was limited to the ten years 1977, 1984, 1998, 2002, 2004, 2007, 2010, 2013, 2016, and 2019. All the orthoimages were resampled to 1 m of pixel size. The SegOptim R package (Gonçalves et al., 2019) was applied to run a segmentation and turn the pixels into objects using Object Based Image Analysis (OBIA) technique, and then to classify the orthoimages through a random forests algorithm, with mean, standard deviation and first and third quartiles as classification features. Each orthoimage was classified into three cover classes: Shrubs (Class 1), forest canopies (Class 2), and bare ground or grasslands (Class 3). A minimum of 70 training plots per cover class and per year were selected by photointerpretation and used to assess classification accuracy (confusion matrixes) using the SegOptim package. The time series of classified orthoimages were used to quantify cover changes (rate of canopy loss and recovery) through time at each of the three defined altitudinal bands.

## 3 Results

### 3.1 Climatic data and field survey

Both mean temperature and radiation data have shown a rising tendency in the last decades, especially since the 1970s (**Supplementary Figures 2A, B**). Drought intensity has also increased, especially after 2002. The last four years of the time series (2017-2020) had the lowest SPEI values, indicating a recent tendency to even more intense drought spells (**Supplementary Figure 2C**).

**Figure 2:**
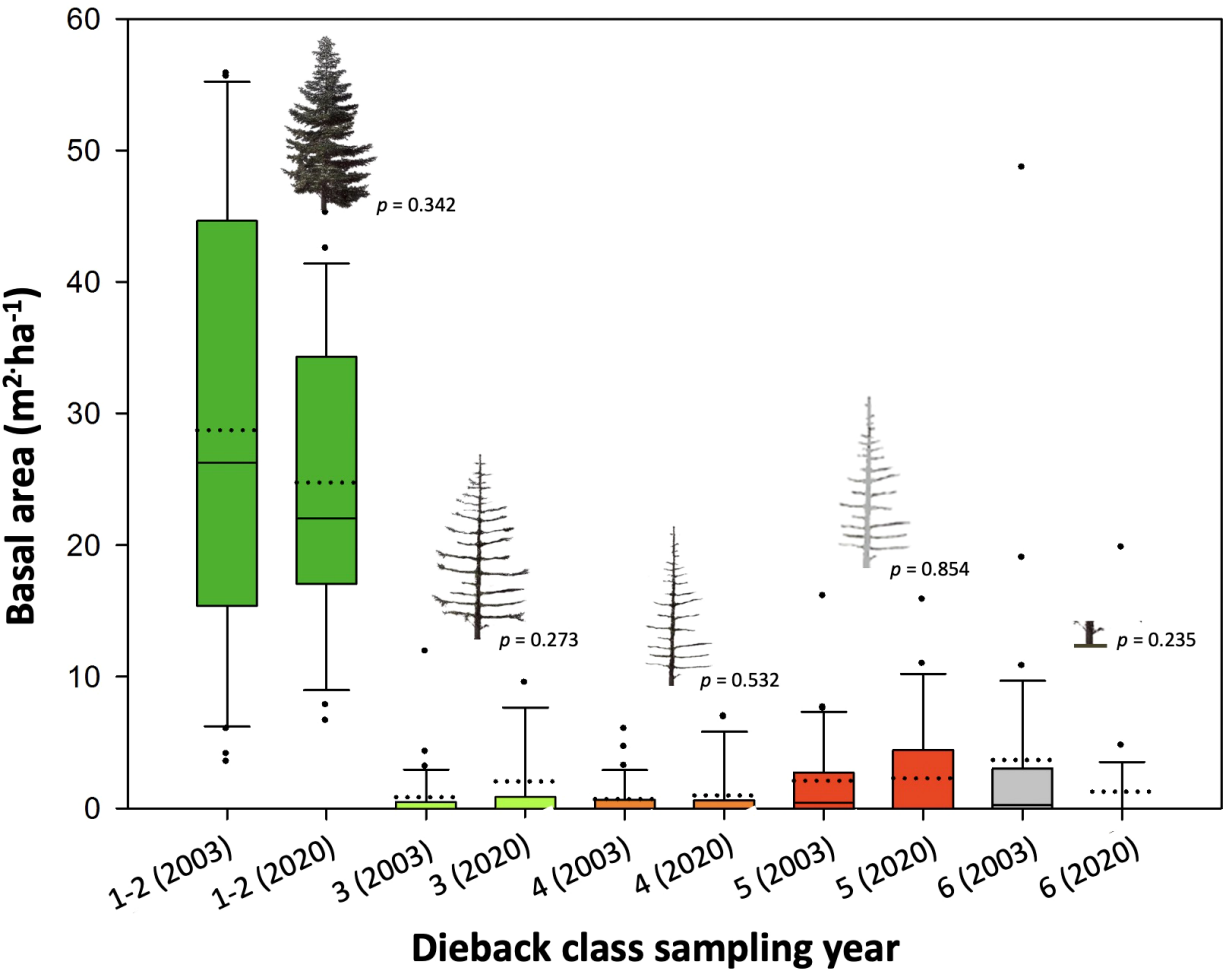
Total basal areas for the different tree classes, as obtained in the 2003 and 2020 field surveys performed on thirty-one *A. pinsapo* stands. Alive (tree-class 1); declining (class 2); and dead trees (classes 3 to 6 ) were classified according to canopy dieback (defoliation, needles brownness) and bark/wood decay symptoms (see also **Supplementary Figure 1**). An ANOVA was performed to compare the basal areas between years within damage class. *P*-values are indicated for each comparison (see also **Supplementary Table 1**).

**Figure 3:**
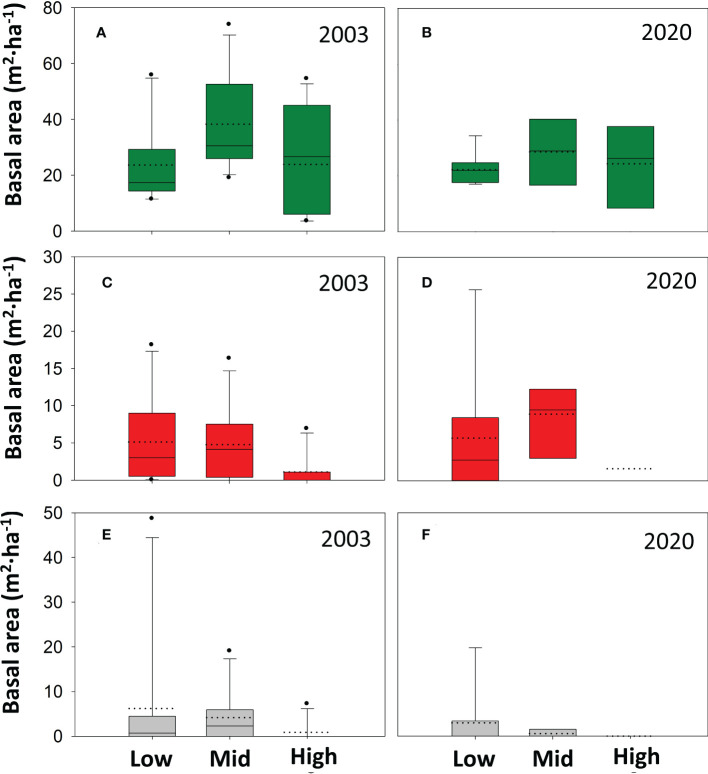
Altitudinal distribution in 2003 and 2020 of the basal area of *A. pinsapo* tree classes. Altitudinal bands: Low (880-1150 m a.s.l.), Mid (1150-1350 m), and High (1350-1550 m). Tree classes: alive trees (in green, **A** and **B**), dead trees (in red, **C** and **D**), and stumps (in grey, **E** and **F**). The ANOVA tests only showed significant increase in mortality between the 2003 high altitude band and the 2020 mid altitude band (**Supplementary Table 2**).

Across altitudinal bands, the average total *A. pinsapo* basal area was 36.0 m^2^ ha^-1^ in the 2003 field survey, and still alive trees (dieback classes 1, and 2), dead trees (classes 3, 4, and 5) and stumps (class 6) accounted for 79.7, 10.0, and 10.3% of the total basal area (**Figure 2**). The basal area accounted for each tree-class had not significantly changed almost two decades later (*p* > 0.05, **Supplementary Table 1**). In 2020, dead trees and stumps still accounted for more than 20% of the total basal area. Alive trees (classes 1 and 2) together remained the dominant ones in the population, and the basal area of more recently dead trees (classes 3-4) showed values like those observed in 2003 (**Figure 2**). Alive trees showed a higher basal area at mid than at low and high altitudes both in 2003 and 2020 (**Figure 3**). Meanwhile, declining and dead trees had also not changed between these two sampling years (**Supplementary Table 2**). Mortality only was higher in the 2020 mid elevation compared to the 2003 high elevation band.

### 3.2 Dynamic factor analysis

**Figure 4:**
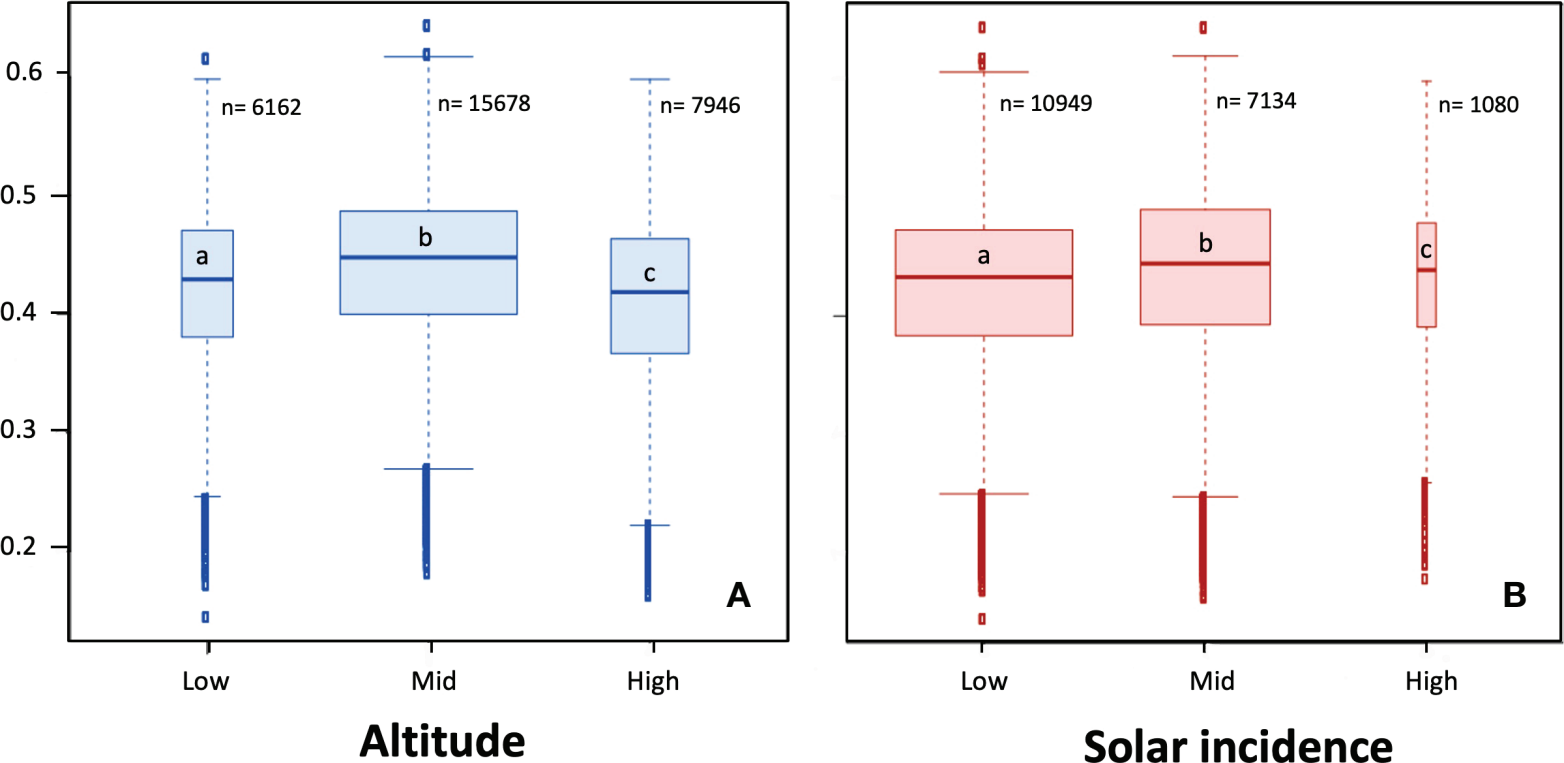
NDVI of altitudinal bands **(A)** and solar incidence value ranges **(B)**. Different letters indicate significant differences between altitude or solar incidence levels (*p* < 0.05; Bonferroni *post-hoc* tests following repeated measures ANOVA; see **Supplementary Table 3**). N values correspond to the number of pixels for each band.

NDVI values for the different altitudinal bands and SI (solar radiation incidence) levels were mainly comprised between 0.4 and 0.5 (**Figure 4**). The repeated measures ANOVA indicated significant effects (*p* < 0.05) of altitude and solar incidence on absolute NDVI values (**Supplementary Table 3**), although the effect sizes were small. A Bonferroni test confirmed significant differences between all levels of both factors (*p* < 0.05). The SI value range with the least number of pixels was the one corresponding to high solar incidence (130-175 h; 1080 pixels). Pixels with a low solar incidence (0-46 h) were the most abundant (10949 pixels). Mid altitude belt (1150-1350 m) was the most abundant in the NDVI data (15678 pixels).

**Figure 5:**
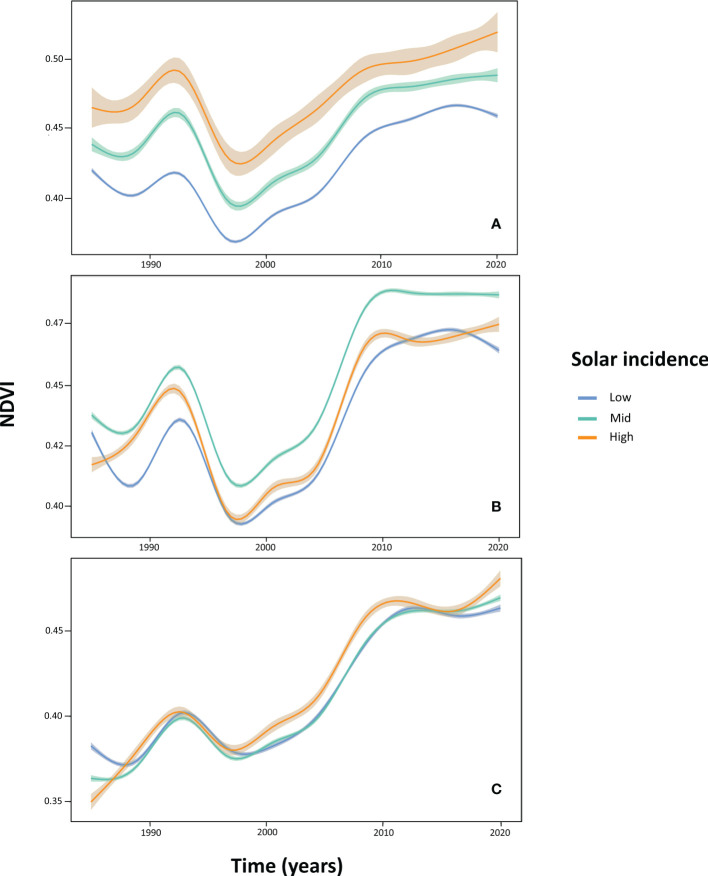
NDVI dynamics at the low (880-1150 m; graph **A**), mid (1150-1350 m; graph **B**), and high (1350-1550 m; graph **C**) altitudinal bands, depending on November solar radiation incidence-SI levels (‘Low’ level: in blue, 0-46 hours of solar incidence; ‘Mid’: in green, 46-130 hours; ‘High’: in red, 130-175 hours). GAM smoothing was applied to NDVI curves, and shaded areas represent the amount of variation in NDVI values (95% confidence intervals).

The temporal pattern of change in NDVI values differed depending on altitude and SI (**Figure 5**). In the low altitudinal band (880-1150 m), NDVI becomes modulated by SI: the higher the SI range pixels belong to, the higher their NDVI values, irrespective of the considered year. However, at mid altitudes (1150-1350 m), higher NDVI values were found in areas subjected to intermediate levels of SI (46-130 h) in all years. The highest altitudinal belt (1350-1550 m) showed no distinct patterns of NDVI dynamics among SI value ranges.

**Figure 6:**
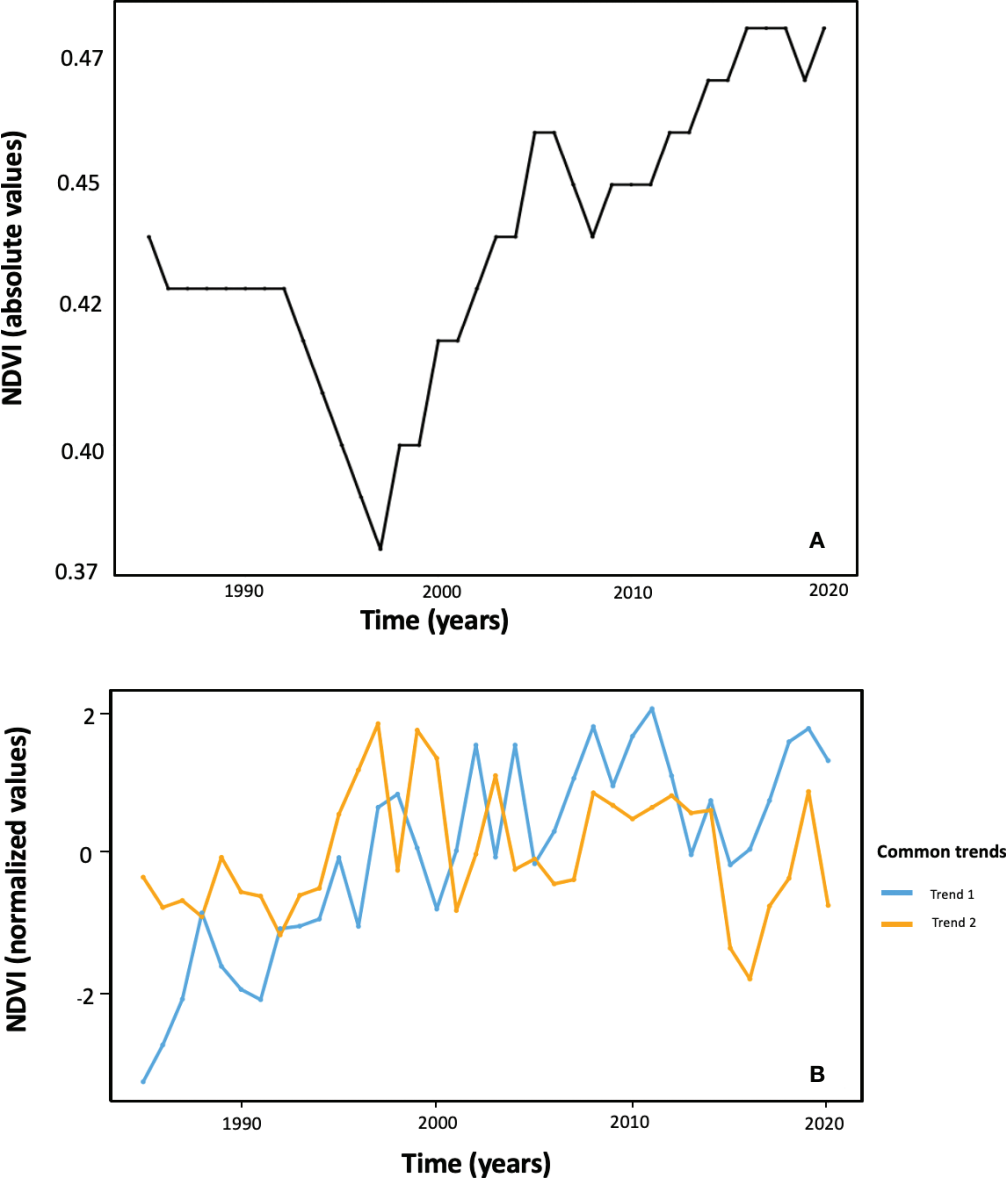
Common temporal trends among the nine NDVI time series corresponding to the different altitude by solar incidence combinations, as revealed by Dynamic Factor Analysis (DFA) performed both on absolute (graph **A**) and on normalized (graph **B**) data.

Dynamic Factor Analysis (DFA) performed on NDVI time series expressed in absolute value revealed just one single significant common temporal trend (AIC = -2050.05) between the nine altitudes by SI combinations (**Figures 6A**; **Supplementary Table 4**). Such a common trend highlights the generality over the studied area (i) a drop in NDVI values from the mid-1990s; (ii) a subsequent rapid recovery along the 2000s (but with a partial drop by the end of this decade) that reaches NDVI levels similar to those observed at the beginning of the time series; (iii) a further increasing trend from 2010 but at a lower rate; and (iv) a trend of stabilization in the last years at NDVI levels that are the highest of the whole time series.

Two common trends (AIC = 324.298) were obtained from the DFA applied to normalized NDVI values. This analysis reveals the patterns of high frequency (successive up and down pulses) in detrended NDVI time series (**Figure 6B**). The common trend 1 was correlated with summer and annual radiation (Rsu = 0.65, Rye = 0.54, respectively) and describes an undulating increasing tendency which tends to stabilize in the last decade of the time series. The common trend 2 showed no correlation with climate variables, and no long-term trend but mid frequency ample pulses. Contrary to the case of the DFA on absolute NDVI values, the different ‘altitude by SI’ combinations do show varying contributions to the two common trends extracted from normalized NDVI series. The high altitude and SI NDVI time series shows a high and positive factor loading on the first common trend, whereas the times series ‘NDVI_1150_175’, ‘NDVI_1350_130’, and ‘NDVI_1550_46’ are the ones with more contribution on common trend 2 (**Supplementary Table 4**).

### 3.3 Linear mixed effect models

Low- and high-elevation showed similar contributions to the variance (33% and 36%), while the explained variance was lower at mid elevation (24%). At low-elevation, the total radiation of the autumn of the previous year was the most significant variable (positive correlation, 29% of relative weight), followed by SI (positive correlation, 23% of relative weight).

At mid elevation, the total radiation of the autumn of the previous year was also the most significant variable (positive correlation, 31% of relative weight in the model), followed by the spring drought index (positive correlation, 20% of relative weight in the model) and the summer drought index (negative correlation, 19% of relative weight in the model). The SI also showed a significant effect, as well as the temperatures of spring and summer (all of them with positive correlations with NDVI).

Contrasting to low and mid, the high elevation belt did not show a significant effect on the SI. The total radiation of the autumn of the previous year and the spring drought index were significant (both with positive correlation, 25% of relative weight, **Tables 1**, **2**).

**Table 1:** Model selection criteria for *Abies pinsapo*-dominated forests NDVI along three elevation belts: low elevation (880-1150 m a.s.l.), mid elevation (1150-1350 m a.s.l.) and high elevation (1350-1550 m a.s.l.).

Fixed factors	K	ΔAICc	*L*	*W*i	*W*1/*W*i
Low Elevation (n=193)					
SI+Raup+Rsu+Rwi+Tsp+Tsu+Ssp+Ssu	10	0	1	0.26	
SI+Raup+Rsu+Tsp+Tsu+Ssp+Ssu	9	0.04	0.98	0.25	98.17
SI+Raup+Rsu+Tsu+Ssp+Ssu	8	0.21	0.9	0.23	91.7
**SI+Raup+Tsu+Ssp+Ssu**	7	0.55	0.76	0.2	84.53
SI+Raup+Ssp+Ssu	6	3.27	0.19	0.05	25.6
SI+Raup+Ssu	5	6.76	0.03	0.01	17.45
Raup+Ssu	4	11.56	0	0	9.1
Raup	3	19.38	0	0	2.01
null model	2	27.5	0	0	1.72
Sum			3.87		
Mid Elevation (n=210)					
SI+Raup+Rsu+Rwi+Tsp+Tsu+Ssp+Ssu	10	0	1	0.37	
SI+Raup+Rsu+Tsp+Tsu+Ssp+Ssu	9	0.17	0.92	0.34	92.04
**SI+Raup+Tsp+Tsu+Ssp+Ssu**	8	0.74	0.69	0.26	75.02
SI+Raup+Ssp+Ssu	6	5.28	0.07	0.03	10.32
Raup+Ssp+Ssu	5	9.02	0.01	0	15.47
Raup+Ssu	4	18.46	0	0	0.89
Raup	3	43.27	0	0	0
null model	2	69.75	0	0	0
SI+Raup+Tsu+Ssp+Ssu	7	715.95	0	0	0
Sum			2.69		
High Elevation (n=133)					
SI+Raup+Rsp+Rwi+Tsp+Tsu+Ssp+Ssu	10	0	1	0.31	
SI+Raup+Rsp+Tsp+Tsu+Ssp+Ssu	9	0.07	0.97	0.3	96.54
Raup+Rsp+Tsp+Tsu+Ssp+Ssu	8	0.46	0.79	0.24	82.24
**Raup+Rsp+Tsp+Ssp+Ssu**	7	1.73	0.42	0.13	53.01
Raup+Tsp+Ssp+Ssu	6	4.92	0.09	0.03	20.31
Raup+Ssp+Ssu	5	11.08	0	0	4.6
Raup+Ssu	4	17.09	0	0	4.96
Raup	3	35.77	0	0	0.01
null model	2	55.88	0	0	0
Sum			3.27		

Seasonal data of temperature (T), Radiation (R) and SPEI (S), as well as site solar incidence (SI), were tested as fixed factors, while sampling points were included as random factors. Seasons are noted as: aup, prior autumn; wi, winter; sp, spring; su, summer; autumn of the current year were not included as the NDVI data were obtained for August-September. A null model assuming NDVI constant was also tested. The K column represents the number of parameters included in the model, that is, the number of explaining variables plus one constant plus the error. Models in bold correspond to models with substantial support. ΔAICc is the difference Akaike information criterion with respect to the best model. *L* is the likelihood of a model given the observed NDVI data. *W*i is the relative probability that the model i is the best model for the observed data, given the candidate set of models. *W*1/*W*i is the evidence ratio percentage for each model with each other model. N is the number of sampling points.

**Table 2:** Outputs of the best-supported models showed in **Tables 1**.

Model			Variable	Value	Std. Error	t-value	Relative weight (%)
Model_L_NDVI=lme(NDVI~si+Raup+Tsu+Ssp+Ssu, method= ‘REML’, random=~1|x)			Intercept	-0.33	7.99·10^-3^	-41.8	
			SI	5.07·10^-4^	7.01·10^-6^	72.4	23.3
			Raup	7.39·10^-4^	8.21·10^-6^	90.0	28.9
			Tsu	0.02	3.88·10^-4^	52.7	16.9
AIC		BIC	Ssp	8.48·10^-3^	1.58·10^-4^	53.4	17.2
-314825.5		-314749.2	Ssu	-7.40·10^-3^	1.74·10^-4^	-42.7	13.7
Model_M_NDVI=lme(NDVI~si+Raup+Tsp+Tsu+Ssp+Ssu, method= ‘REML’, random=~1|x))			Intercept	-0.21	4.60·10^-2^	-46.0	
			SI	1.54·10^-4^	2.50·10^-6^	61.8	13.1
			Raup	7.31·10^-4^	4.91·10^-6^	148.8	31.4
			Tsp	8.62·10^-3^	3.18·10^-4^	27.0	5.7
			Tsu	1.19·10^-3^	2.35·10^-4^	50.4	10.6
AIC		BIC	Ssp	9.18·10^-3^	9.74·10^-5^	94.2	19.9
-1030031.0		-1029934.0	Ssu	-9.31·10^-3^	1.01·10^-4^	-91.5	19.3
Model_H_NDVI=lme(NDVI~Raup+Rsp+Tsp+Ssp+Ssu, method="REML", random=~1|x)			Intercept	-0.54	5.96·10^-3^	-91.1	
			Raup	7.80·10^-4^	7.64·10^-6^	102.0	23.6
			Rsp	4.50·10^-4^	7.92·10^-6^	56.9	13.2
			Tsp	0.03	4.50·10^-4^	67.3	15.5
AIC	BIC		Ssp	0.02	1.75·10^-4^	114.6	26.5
-465951.5	-465872.0		Ssu	-0.01	1.4·10^-4^	-92.1	21.3

Model_L_NDVI: Low elevation best supported model; Model_M_NDVI: Mid elevation best supported model; Model_H_NDVI: High elevation best supported model. The degrees of freedom were 102006, 347008 and 154194, for low, mid, and high elevation, respectively; *p*-values were < 1·10^-15^ in all cases. The relative weights of the selected environmental variables related to NDVI were computed excluding the intercept.

**Figure 7:**
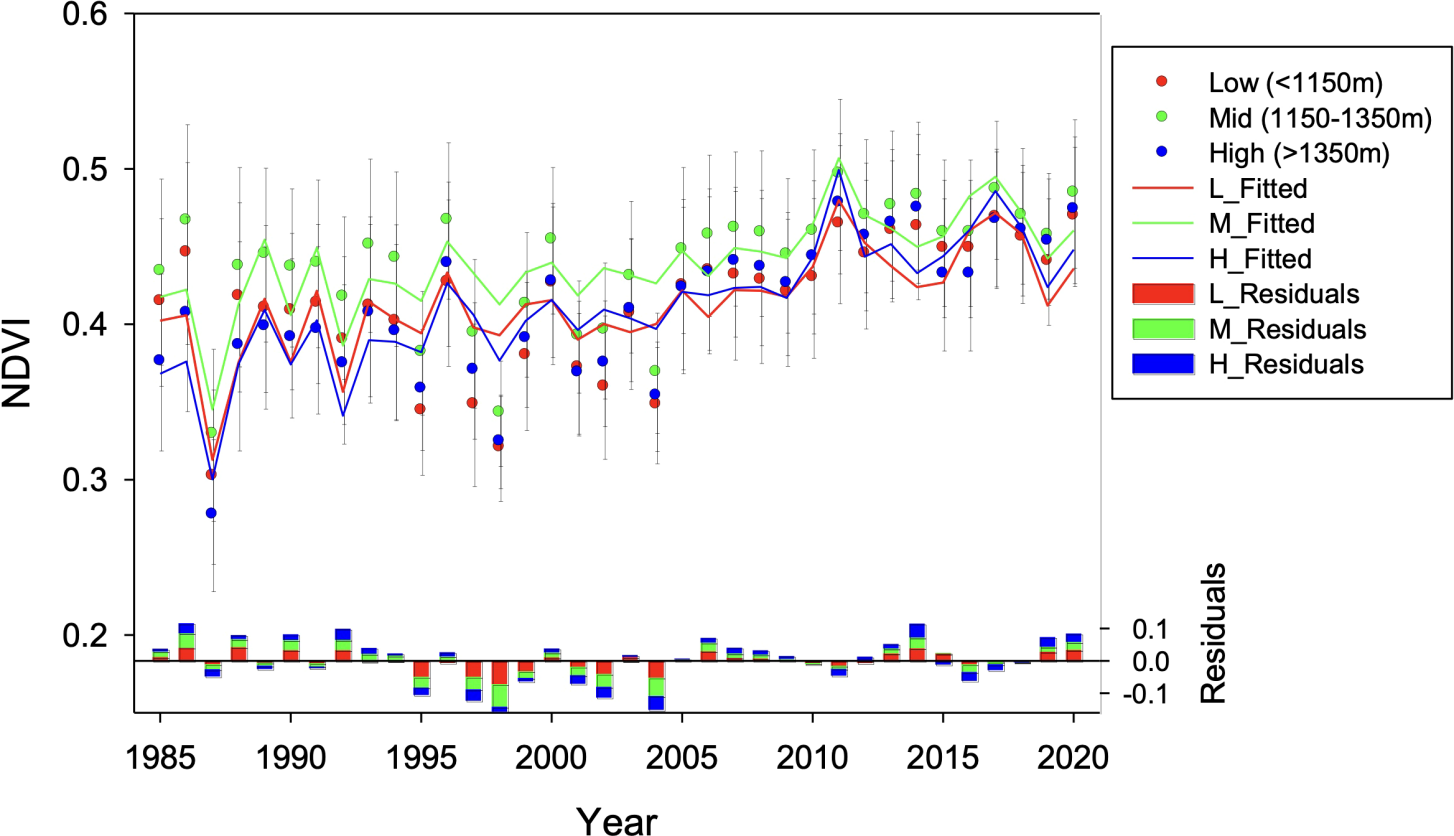
Predicted NDVI obtained by linear mixed effects models (LMEM) using climate and physiography as predictors. NDVI was fitted for low (880-1150 m a.s.l.; L_Fitted in red), mid (1150-1350 m a.s.l.; M_Fitted in green), and high (1350-1550 m a.s.l.; H_Fitted in blue) altitudinal bands. The residuals of the models (bottom bars) were computed as the difference between observed and predicted NDVI; positive bars denote years where observed NDVI values were higher than expected based on climate, while negative bars denote years where observed NDVI values were lower than expected based on climate.

The residuals of the models were not related to elevation (**Supplementary Figure 3**) and did not show time autocorrelation. However, the residuals were markedly negative (that is, the predictions are systematically above the observations) between the years 1995-2006 at low and middle elevations, and between the years 1995-2004 at higher elevations (**Figure 7**; **Supplementary Figure 4**).

### 3.4 Canopy loss evolution

The classification of orthoimages into three land-cover classes (shrubs, forest canopy, bare ground-grasslands) achieved an accuracy between 0.8-0.9 and the Kappa index ranged from 0.56-0.86 (**Supplementary Table 5**). The percentage of pixels classified as canopy (**Supplementary Figure 5**) showed a drop in the 1998 orthoimage, while it strongly rose in the 2002 and 2004 images. Since then, the forest canopy coverage has remained stable except for a moderate fall in the 2013 orthoimage.

**Figure 8:**
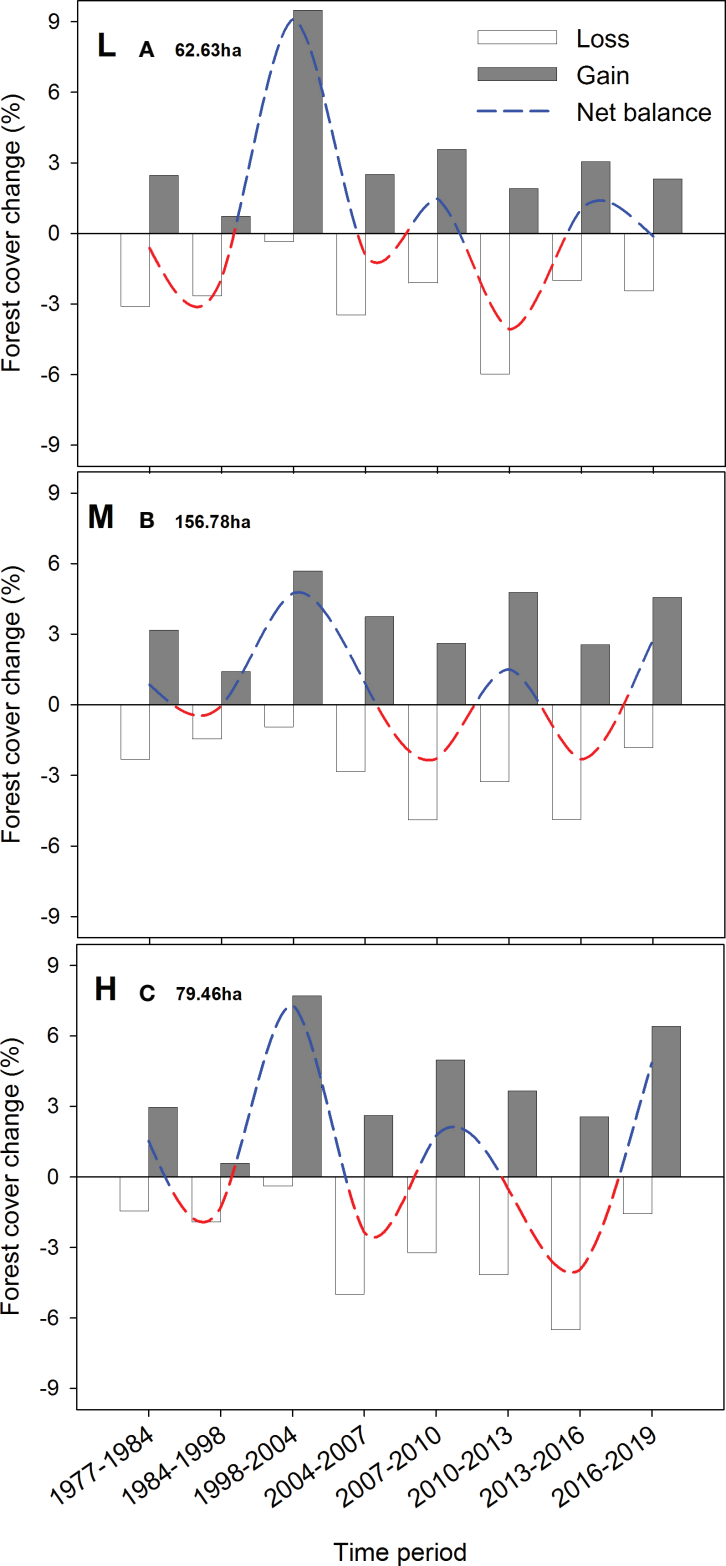
Annual rate of forest cover change is estimated as the loss (empty bars) and gain (gray bars) of forest surface (ha) between the dates of sequential aerial orthophotographs, divided by the number of years included in that period. The net balance was estimated as the difference between gains and losses in each period, depicting forest surface increases (blue dashed line) or decreases (red dashed line). Estimates were performed for low (880-1150 m a.s.l.; graph **A**), mid (1150-1350 m a.s.l.; graph **B**), and high (1350-1550 m a.s.l.; graph **C**) altitudinal bands. The total area (ha) for each band is also shown to understand the scale of the forest cover change.

Rates of forest cover change showing canopy loss (transitions from forest to shrubs or bare ground/grassland covers) between two orthoimages were the highest at the mid altitudinal band (1150-1350m) during 2007-2010 and 2013-2016 (> 7 ha·year^-1^). However, this band is the most abundant in the study area (>150 ha), so it also was the band that experienced the highest recovery rate (**Figure 8**). All bands coincide in a negative net balance for the period (1984-98), with the mid band being the one least affected. The highest canopy recovery rate was dated for the 1998-2004 period in all altitudes (6-9 ha·year^-1^).

Later on (2004-2019), acute peaks of canopy loss (3-8 ha·year^-1^) showed asynchrony across elevations. This loss was subsequently compensated by events of canopy gaining. However, in the last years (2016-2019 comparison), all bands coincided with an increase in the canopy gain.

## 4 Discussion

**Figure 9:**
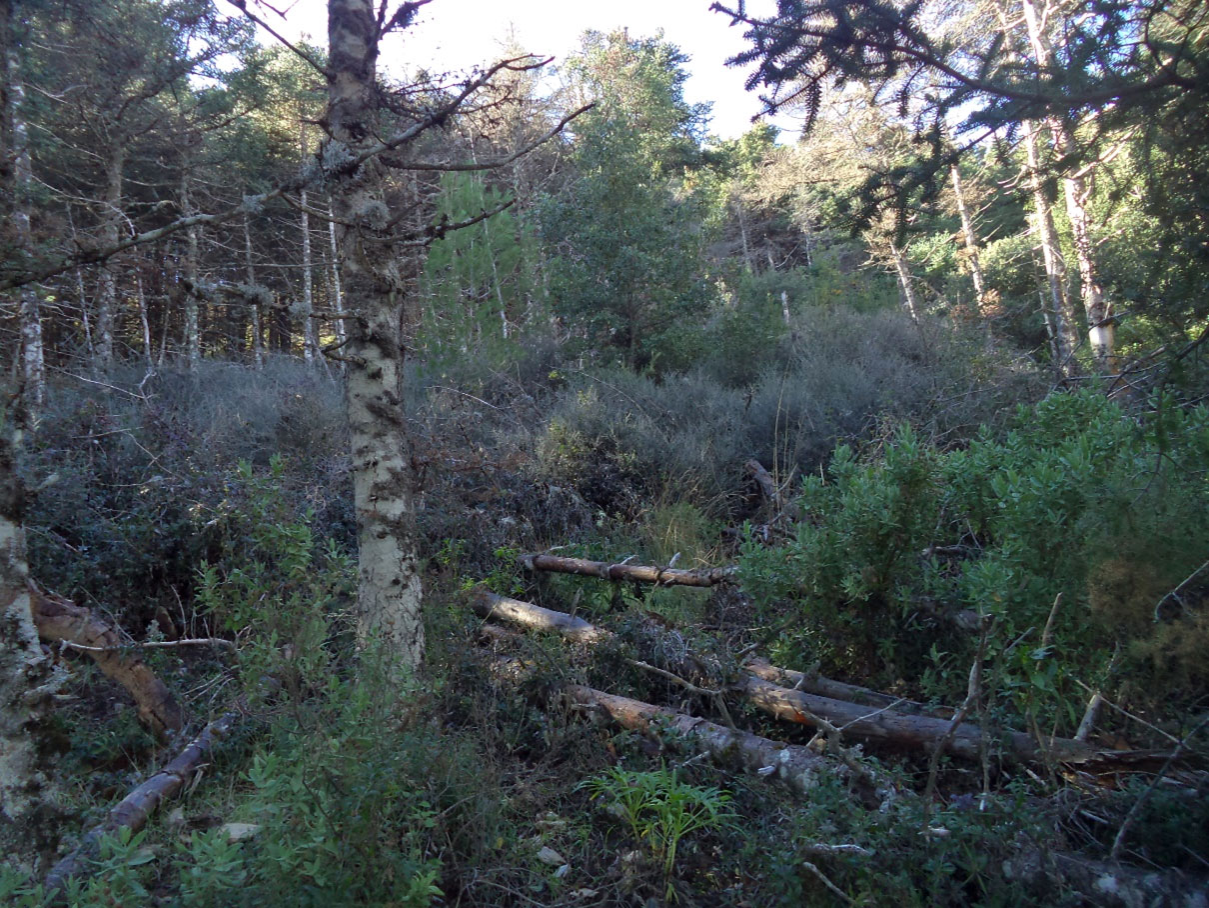
Forest gap caused by the dieback process and its recolonization and vegetation. Picture taken in the 2020 field survey.

*Abies pinsapo* is known to have traits that suggest its capability to cope with Mediterranean summer conditions even in the absence of phenophasical plasticity (Latorre and Cabezudo, 2012). However, previous studies (Linares et al., 2011) indicated that, compared with other coexisting tree species, *A. pinsapo* shows a limited drought adaptive capacity in its main distribution area. For instance, in contrast with Pinus halepensis Mill. individuals in the same stands, *A. pinsapo* ones showed more acute growth reduction trends in the last decades, sudden growth reductions, and less responsive water use efficiency in the face of drought spells (Linares et al., 2011). In fact, severe symptoms of stand stagnation and forest decline during the last decades, associated with regional trends of climate aridification, have been reported in some of the most important *A. pinsapo* populations (Linares et al., 2009c). These effects are synergistically amplified by increasing levels of intraspecific competition linked to rural abandonment and strict conservation policies that led to an absence of low intensity disturbances -no forest management nor herbivore activity- (Linares et al., 2010a). The consequence has been an extensive forest dieback and gap opening process (**Figure 9**; **Supplementary Figure 6**), with the severe 1994-95 drought acting as a tipping point, so that very bad prospects had been forecasted for *A. pinsapo* (e.g., rapid rear-edge retraction, one of the highest vulnerabilities to climate change among circum-Mediterranean fir species; Linares et al., 2010a; Sánchez-Salguero et al., 2017).

Severe decline and die-back processes for other forest species in the Mediterranean region have been reported (e.g., Camarero et al., 2018). Drought and heat-induced tree mortality are currently ubiquitous worldwide, affecting forest biomes from temperate to tropical regions (Allen et al., 2010). Moreover, it has been proposed that current knowledge and models might even be underestimating global forest vulnerability to climate change, so the impact could be even worse (Allen et al., 2015).

In this study, we observe similar climatic trends of rising temperature (**Supplementary Figure 2A**) and aridification (**Supplementary Figure 2C**, SPEI), which have worsened compared to previous research based on times series ending in 2005 (Linares et al., 2009a; Linares et al., 2011). The 2003 versus 2020 field-survey data demonstrate also the persistence of high mortality rates nowadays (tree classes 4 to 6 accounted for over 20% of the stands basal area in 2020; **Figure 2**). These observations are congruent with recent reports of the *A. pinsapo* dieback process based on field survey data (Navarro-Cerrillo et al., 2022).

However, the multiscale approach and longer time frame since initial mortality events applied in the present work has yielded results that indicate an unexpected resilience of *A. pinsapo* forests to dieback, thus not confirming the bad prospects forecasted by previous studies. Nevertheless, observations of recovery trends in *A. pinsapo* populations have been reported by Gutiérrez-Hernández et al. (2018), who also described an increasing tendency of NDVI values in *A. pinsapo* forests and an earlier start of the green up.

In our study, the fact that predicted NDVI based solely on climatic data is systematically above observations from the mid-1990s to mid-2000s (**Figure 7**) can be explained by concomitant high mortality (but still low recovery). However, NDVI time series indicate a sharp recovery of forest cover and biomass in the late 2000s, and stable levels in the last decade which are maximums within the whole 1984-2020 time series, at all altitudes independently of SI (**Figure 5**). Similarly, multi-temporal analysis of classified orthoimages showed a synchronic maximum of canopy gain across altitudes for the 1998 *vs* 2004 comparison (**Figure 8**). The NDVI returned to be adequately modeled by climatic conditions in the LMEM analysis during the last decade of the studied time series (**Figure 7**).

Ecologists are generally inclined to expect regime shifts when studying community dynamics against disturbance, since the latter is considered when it causes visible changes in biomass or species density and composition (Connell & Ghedini, 2015). However, there is increasing evidence of somewhat hidden compensatory mechanisms, triggered early as short-term responses, that adjust the system dynamics to counter the otherwise unchecked effects of disturbance, which are important in explaining apparently unexpected long-term processes of recovery (Lloret et al., 2012; Loreau & de Mazancourt, 2013). In our study case, dieback is acting as a continuous background process that opens forest gaps. However, the present net balance in *A. pinsapo* forest status seems positive, which may have resulted from the following compensation phenomena that might be involved: (i) recruitment of new *A. pinsapo* individuals; (ii) facilitative effects on such recruitment mediated by revegetation with other tree species and shrub species; and (iii) a ‘release effect’ in which surviving *A. pinsapo* trees can thrive with fewer resource competition.

Several of our results support this compensation phenomena hypothesis. First, the mean alive *A. pinsapo* basal area was not significantly different between the 2003 and the 2020 field surveys (**Figure 3A, B**). Second, by the end of the considered time frame, NDVI values were maximum (> 0.5) at the lowest altitudinal band and in positions with the highest SI values (**Figure 5A**). Low altitude and high irradiance mean drier conditions, which might have promoted the colonization of mortality gaps by thermophile vegetation (*Pinus halepensis, Juniperus* sp, *Cistus* sp, *Ulex* sp), through dispersal from below the *A. pinsapo* lower ecotone. Mortality gap invasion by thermophile shrubs has been recently evidenced in the area through the characterization of recent changes in fuel models (vegetation structure) in a study on fire risks (Cortés-Molino et al., 2020). Encroachment of thermophile shrubs and bushes in open gaps would facilitate the understory establishment of shade-tolerant *A. pinsapo* saplings, whose growth would over time exceed the height of the bushes. Third, the recovery of NDVI values during the mid-1990s and 2000s showed steeper slopes at mid and high altitudes than at low altitude (**Figure 5B**), which suggest a stronger contribution of the ‘release effect’ at the former elevations. This steeper slope in NDVI is especially clear for the mid altitudinal band, which showed a rapid response in less than a decade (~2005-2010). Consequently, the aforementioned gap recolonization process should not have time enough to fully develop at mid altitudes. This band was also the one with the least negative canopy balance (<1 ha· year -1; **Figure 8**) during the 1984-98 period. Evidence of growing crowns can be observed from aerial orthoimages (**Supplementary Figure 7**). The combination of all these three effects could explain the rebound effect in NDVI following the 1994-95 drought. However, compensation mechanisms have recently been shown to commonly fail in marginal tree populations in the long term, as has been reported in a study comprising more than fifty North American tree species (Yang et al., 2022).

Other effects, such as CO_2_ fertilization should not be discarded. Indeed, vegetation greenness has been increasing globally, at least since the early 1980s (Piao et al., 2020). Despite vegetation models suggest that CO_2_ fertilization is the main driver of greening on the global scale, other factors might be significant at the regional scale. In our case, further research is needed to disentangle the extent to which the observed rising NDVI (**Figure 7**) also indicates an increasing carbon sink. Rising atmospheric N deposition may be also hypothesized a process involved in the increasing NDVI (Mao et al., 2012). Notwithstanding, this explanation lacks substantial support in our study site, as these *A. pinsapo* stands present limited N deposition (Blanes et al., 2013).

This positive recovery alleviates initial warnings about the dieback process in an endemic species with high conservation value. However, this resilience also implies risks derived from side effects that should be accounted for in management plans. The increasing fire risk due to the rising flammability of fuel models associated with the vegetation recovery dynamics is particularly relevant (Cortés-Molino et al., 2020). At the local scale, dead wood accumulation in recent mortality gaps, and fuel structures with overlap between shrubs, bushes, and tree crowns in revegetating gaps, increase flammability and spread probability. These changes in the fuel models highlight the need for a strategy of proactive management to limit fire risks; otherwise, the good news of high resiliency of *A. pinsapo* forest to drought-induced dieback might turn into bad news of wildfires. It has been demonstrated that the Spanish fir shows no resilience to wildfires, being the worst threat to their populations (Silva, 1996). This message is also valid for other tree species with wider distribution, which are relevant in terms of economic value and ecosystem services provision such as *Abies cephalonica Loud*. (Ganatsas et al., 2012) or *Cedrus atlantica Endl.* (Abel-Schaad et al., 2018).

We might be underestimating the resilience of the circum-Mediterranean firs to climate change, despite wide evidence about their drought sensitivity (Sánchez-Salguero et al., 2017). Indeed, recent studies suggest that silver fir (*Abies alba* Mill.) can grow and regenerate under Mediterranean conditions, forming mixed stands with evergreen (e.g., *Quercus ilex* L.) and deciduous Mediterranean tree species, while its drought sensitivity was also stated (Walder et al., 2021).

## 5 Conclusion

Despite decades of persistent drought-induced decline and mortality in *A. pinsapo* forests, the results presented here, based on multitemporal and multiscale data from field surveys, orthoimages, and satellite imagery showed: (i) a rising photosynthetic activity since mid-2000s (ii) an almost steady alive basal area of *A. pinsapo* stands between 2003 and 2020 and (iii) an increase in forest canopy gains in the last years at mid and high elevation bands.

These results support the hypothesis of an *A. pinsapo* forests resilience to climate change events, perhaps, greater than expected. However, further research is needed to understand the scope of these compensation mechanisms, which other processes could be involved in this recovery and what role will they play in future climate change scenarios.

## Data availability statement

The original contributions presented in the study are included in the article/**Supplementary material**. Further inquiries can be directed to the corresponding author.

## Author contributions

AC-M performed the experiments, analyzed the data, prepared figures and tables, authored and reviewed drafts of the paper, and approved the final draft. JL designed the experiments, performed fieldwork and experiments, analyzed the data, prepared figures, and tables, reviewed drafts of the paper and approved the final draft. BV designed the experiments, analyzed the data, reviewed drafts of the paper, and approved the final draft. VL performed fieldwork and experiments, reviewed drafts of the paper, and approved the final draft. AS-T analyzed the data, authored, and reviewed drafts of the paper and approved final draft. AF-M analyzed the data, authored and reviewed drafts of the paper, and approved the final draft. I-FL analyzed the data, prepared figures and tables, and approved the final draft. JC conceived and designed the experiments, got funding, performed fieldwork and experiments, analyzed the data, authored and fully reviewed drafts of the paper and approved the final draft.
