# Supplementary material for: Unexpected resilience in relict Abies pinsapo Boiss forests to dieback and mortality induced by climate change
Source: Front Plant Sci. 2022 Dec 23;13:991720. doi: 10.3389/fpls.2022.991720 (PMC9822712; doi:10.3389/fpls.2022.991720)
Supplement: Supplementary file 1 [file DataSheet_1.docx]

**-Supplementary materials**

**Table S1.** Results from the ANOVA of tree classes. *P*-values for Year in all classes were > 0.05. (see Fig. 2).

|  | **Class 1_2** | | | | | | | | |
| --- | --- | --- | --- | --- | --- | --- | --- | --- | --- |
| **Factor** | | | **DF** | | **Sum Sq** | **Mean Sq** | **Fvalue** | ***p*-values** |  |
| **Intercept** | | | 1 | | 39234.96 | 9234.96 | 166.26 | < 1·10^-15^ |  |
| **Year** | | | 1 | | 216.61 | 216.61 | 0.92 | 0.342 |  |
| **Error** | | | 53 | | 12742.81 | 235.98 |  |  |  |
|  | **Class 3** | | | | | | | | |
| **Factor** | |  | | **DF Sum Sq. Mean Sq. Fvalue** ***p*-values** | | | | | |
| **Intercept** | |  | | 1 113.77 113.77 7.12 0.010 | | | | | |
| **Year** | |  | | 1 19.54 19.54 1.22 0.273 | | | | | |
| **Error** | |  | | 54 862.18 15.96 | | | | | |
|  | **Class 4** | | | | | | | | |
| **Factor** | |  | | **DF** **Sum Sq. Mean Sq. Fvalue *p*-values** | | | | | |
| **Intercept** | |  | | 1  37.94 37.94 37.94 9.36·10^-4^ | | | | | |
| **Year** | |  | | 1 1.22 1.22 0.39 0.532 | | | | | |
| **Error** | |  | | 54 167.11 3.09 | | | | | |
|  | **Class 5** | | | | | | | | |
| **Factor** | |  | | **DF Sum Sq. Mean Sq. Fvalue *p*-values** | | | | | |
| **Intercept** | |  | | 1 262.17 262.17 17.45 1.08·10^-4^ | | | | | |
| **Year** | |  | | 1 0.50 0.50 0.03 0.854 | | | | | |
| **Error** | |  | | 54 810.96 15.01 | | | | | |
|  | **Class 6** | | | | | | | | |
| **Factor** | |  | | **DF Sum Sq. Mean Sq. Fvalue** ***p*-values** | | | | | |
| **Intercept** | |  | | 1 334.23 334.23 6.06 1.08·10^-4^ | | | | | |
| **Year** | |  | | 1 79.18 79.18 1.43 0.234 | | | | | |
| **Error** | |  | | 54 2976.23 55.11 | | | | | |

**Table S2.** Results from the ANOVAs performed on BAI per altitudinal bands, obtained from the two surveys -2003 and 2020- (see Fig. 3). Tukey post-hoc test only showed significant higher mortality in 2020 mid elevation band comparing to 2003 high elevation band (*p*-value = 0.042).

| **Alive trees** |  |  |  | |  |  |
| --- | --- | --- | --- | --- | --- | --- |
| **Factor** | **DF** | **Sum Sq** | **Mean Sq** | | **Fvalue** | ***p*-values** |
| Intercept | 1 | 39128.72 | 39128.72 | 176.85 | | - |
| Year | 5 | 1896.94 | 379.39 | 1.71 | | 0.148 |
| Error | 50 | 11062.49 | 221.25 | - | | - |
| **Dead trees** |  |  |  |  | |  |
| Intercept | 1 | 1126.86 | 1126.86 | 39.22 | | - |
| Year | 5 | 349.93 | 69.78 | 2.49 | | 0.047 |
| Error | 50 | 1436.53 | 28.73 | - | | - |
| **Stumps** |  |  |  |  | |  |
| Intercept | 1 | 328.79 | 328.79 | 5.91 | | 0.018 |
| Year | 5 | 274.97 | 54.99 | 0.98 | | 0.433 |
| Error | 50 | 2780.45 | 55.60 |  | | - |
| Residuals | 603400 | 1305.6 | 0.00 |  | |  |

**Table S3.** Results from the repeated measures ANOVA performed on NDVI time series and Bonferroni pairwise comparison. *P*-values in all factors were < 1·10^-15^, except for the Time*Elevation (2.35·10^-4^) and Time*SI (2.29·10^-7^).

| **Factor** | | | **DF** | **Sum Sq** | **Mean Sq** | | | **Fvalue** | |
| --- | --- | --- | --- | --- | --- | --- | --- | --- | --- |
| Time | | | 35 | 243.2 | 6.95 | 3211.10 | | | |
| Elevation | | | 2 | 86.7 | 43.37 | 20044.77 | | | |
| SI | | | 2 | 33.9 | 16.96 | 7839.37 | | | |
| Elevation*SI | | | 4 | 14.4 | 3.59 | 1659.78 | | | |
| Time*Elevation | | | 70 | 9.5 | 0.14 | 62.96 | | | |
| Time*SI | | | 70 | 7.6 | 0.11 | 49.97 | | | |
| Time*SI*Elevation | | | 140 | 3.0 | 0.02 | 9.91 | | | |
| Residuals | | | 603400 | 1305.6 | 0.00 |  | | | |
| **Pairwise comparison using Bonferroni test** | | | | | | | | |  |
| **NDVI and Elevation *p*-values** | | | | | | | | |  |
| Levels | 1150 | 1350 | | | | |  |  |  |
| 1350 | < 1·10^-15^ | < 1·10^-15^ | | | | |  |  |  |
| 1550 | < 1·10^-15^ | | | | | | | |  |
| **NDVI and SI *p*-values** | | | | | | | | |  |
| Levels | 46 | 130 | | | | |  |  |  |
| 46 | < 1·10^-15^ | < 1·10^-15^ | | | | |  |  |  |
| 175 | < 1·10^-15^ | | | | | | | |  |

**Table S4.** **Factor loadings of the nine “altitude by solar incidence” NDVI time series on the common trends detected by Dynamic Factor Analysis (DFA).** The low, mid and high altitudinal bands are denoted by their upper limits (“1150”, “1350” and “1550”, respectively); the same apply for the solar incidence value ranges (“46”, “130” and “175”, respectively).

| **Time series** | **DFA 1 (absolute NDVI data)** | **DFA 2 (normalized NDVI data)** | |
| --- | --- | --- | --- |
|  | **Trend 1** | **Trend 1** | **Trend 2** |
| NDVI_1150_46 | 0.010 | -0.234 | -0.225 |
| NDVI_1150_130 | 0.010 | -0.112 | -0.185 |
| NDVI_1150_175 | 0.011 | 0.161 | -0.534 |
| NDVI_1350_46 | 0.008 | -0.275 | 0.309 |
| NDVI_1350_130 | 0.007 | 0.014 | 0.478 |
| NDVI_1350_175 | 0.008 | -0.116 | -0.101 |
| NDVI_1550_46 | 0.007 | -0.058 | 0.440 |
| NDVI_1550_130 | 0.006 | 0.138 | -0.007 |
| NDVI_1550_175 | 0.006 | 0.482 | -0.173 |

**Table S5.** Confusion matrixes from the orthoimages classification. Class 1: Shrubs / Class 2: Pinsapo Canopy / Class 3: Bare ground - grassland

| **1977** | | | |
| --- | --- | --- | --- |
| **Predicted** | **Observed** | | |
|  | **1** | **2** | **3** |
| **1** | 409 | 108 | 22 |
| **2** | 161 | 651 | 1 |
| **3** | 18 | 1 | 167 |
| **Kappa** | **PSS** | **GSS** | **Accuracy** |
| 0.6739486 | 0.6739486 | 0.7381568 | 0.8088582 |
| **1998** | | | |
| **Predicted** | **Observed** | | |
|  | **1** | **2** | **3** |
| **1** | 457 | 167 | 16 |
| **2** | 163 | 646 | 0 |
| **3** | 13 | 0 | 25 |
| **Kappa** | **PSS** | **GSS** | **Accuracy** |
| 0.5989007 | 0.5993758 | 0.7033439 | 0.7712775 |
| **2002** | | | |
| **Predicted** | **Observed** | | |
|  | **1** | **2** | **3** |
| **1** | 177 | 98 | 20 |
| **2** | 166 | 952 | 4 |
| **3** | 20 | 1 | 122 |
| **Kappa** | **PSS** | **GSS** | **Accuracy** |
| 0.5893727 | 0.5631768 | 0.6138216 | 0.8102564 |
| **2004** | | | |
| **Predicted** | **Observed** | | |
|  | **1** | **2** | **3** |
| **1** | 143 | 19 | 17 |
| **2** | 51 | 1453 | 7 |
| **3** | 18 | 5 | 106 |
| **Kappa** | **PSS** | **GSS** | **Accuracy** |
| 0.7758212 | 0.7360288 | 0.6968384 | 0.9312914 |
| **2007** | | | |
| **Predicted** | **Observed** | | |
|  | **1** | **2** | **3** |
| **1** | 153 | 17 | 6 |
| **2** | 27 | 1398 | 1 |
| **3** | 8 | 3 | 105 |
| **Kappa** | **PSS** | **GSS** | **Accuracy** |
| 0.8651735 | 0.8582546 | 0.8462251 | 0.9598329 |
| **2010** | | | |
| **Predicted** | **Observed** | | |
|  | **1** | **2** | **3** |
| **1** | 118 | 41 | 6 |
| **2** | 102 | 1304 | 6 |
| **3** | 1 | 4 | 125 |
| **Kappa** | **PSS** | **GSS** | **Accuracy** |
| 0.6894445 | 0.6423380 | 0.6877799 | 0.8980638 |
| **2013** | | | |
| **Predicted** | **Observed** | | |
|  | **1** | **2** | **3** |
| **1** | 179 | 24 | 8 |
| **2** | 59 | 1543 | 1 |
| **3** | 4 | 0 | 146 |
| **Kappa** | **PSS** | **GSS** | **Accuracy** |
| 0.8544705 | 0.8254044 | 0.8342646 | 0.9516280 |
| **2016** | | | |
| **Predicted** | **Observed** | | |
|  | **1** | **2** | **3** |
| **1** | 623 | 130 | 43 |
| **2** | 161 | 2355 | 8 |
| **3** | 18 | 9 | 722 |
| **Kappa** | **PSS** | **GSS** | **Accuracy** |
| 0.8147459 | 0.8084766 | 0.7954032 | 0.8976528 |
| **2019** | | | |
| **Predicted** | **Observed** | | |
|  | **1** | **2** | **3** |
| **1** | 239 | 48 | 18 |
| **2** | 95 | 1405 | 2 |
| **3** | 6 | 0 | 165 |
| **Kappa** | **PSS** | **GSS** | **Accuracy** |
| 0.7909352 | 0.7582873 | 0.7733844 | 0.9155747 |

**
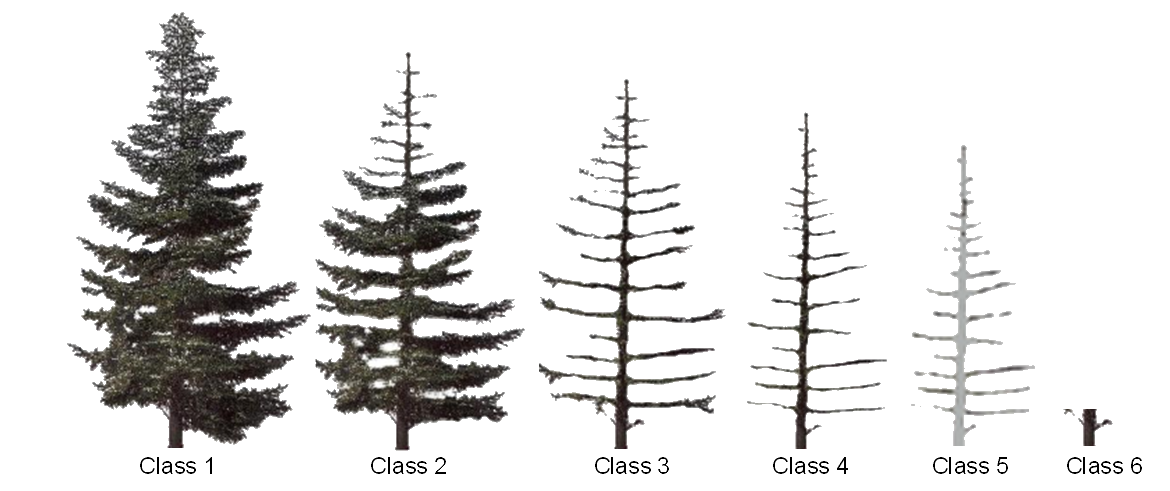
**

**Figure S1.** Dieback classes defined according to defoliation and wood decay states. Class 1 was assigned to healthy trees showing more than 2/3 of the crown green and retaining a significant number of needles cohorts (>5 years old needles; needle longevity reaches up to 10-15 years in *A. pinsapo*). Class 2 corresponds to declining trees showing dieback and severe defoliation in at least 1/3 of the crown; defoliation is very severe in the older needles cohorts (more than five years old needles are almost absent). Class 3 contained trees that died recently (i.e., in the current or the prior year); they are characterized by severe defoliation and brown needles. Class 4 was assigned to trees that died likely 3 - 10 years ago; they are characterized by a total loss of needles and thin branches, while bark is still present in medium and coarse branches and the stem. Class 5 corresponds to trees dead likely more than 10 years ago; they are characterized by a total loss of medium branches and bark is almost absent. Class 6 was assigned to stumps, where species identification was based on bark and wood morphology when possible.

**a)**

**b)**

**c)**

SPEI

**Figure S2.** Climatic trends in the study area showing an increase in solar radiation (a) and, specially, in annual mean temperature (b). The SPEI drought index (c) shows an increasing trend to drier years, with a special dry period in the late years (2018-2020).


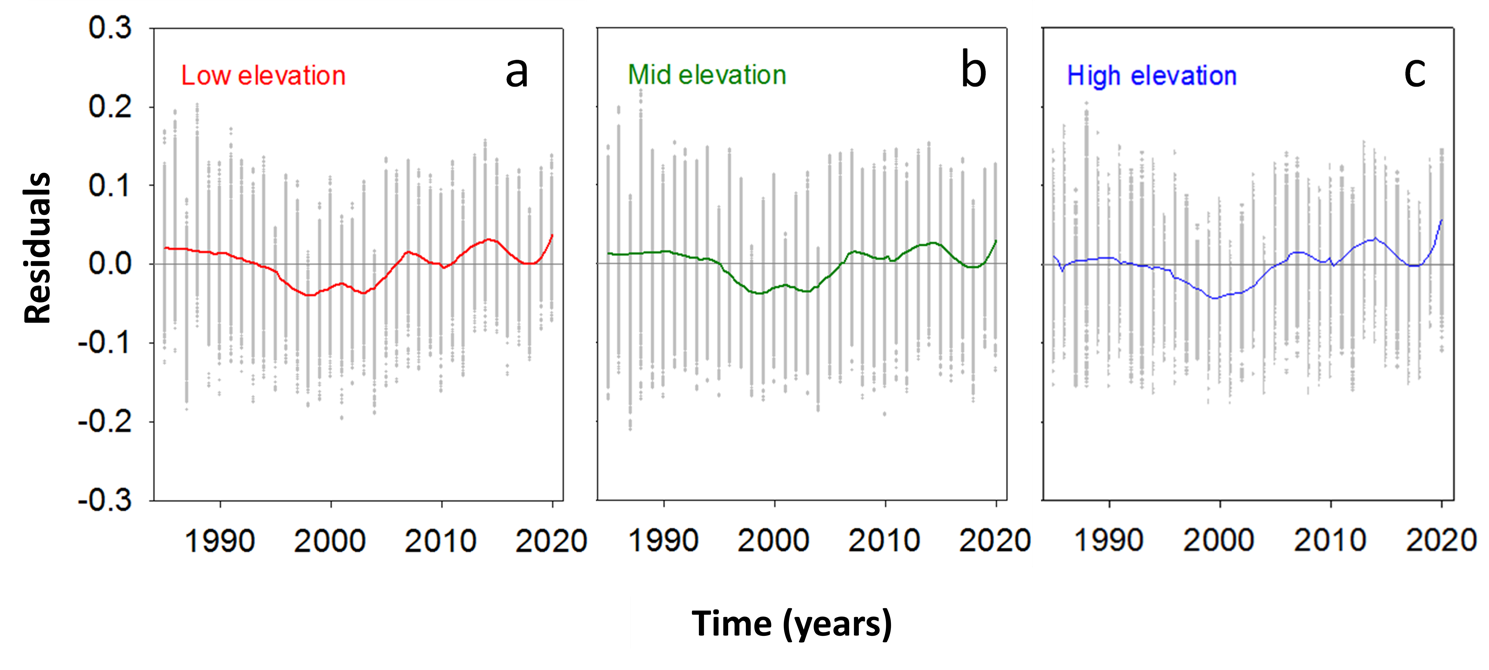


**Figure S3.** Temporal pattern of residuals obtained for 1985-2020 NDVI values al low elevation (a), mid elevation (b) and high elevation (c) of *Abies pinsapo*-dominated forests. Temporal trends were modeled by the Loess smoothing method using a polynomial weight function of degree 3.

**Figure S4**. Model fitting and residuals pattern obtained for 1985-2020 NDVI values al low elevation (left column), mid elevation (central column), and high elevation (right column) of *Abies pinsapo*-dominated forests.

**Figure S5. Temporal change in the percentage of pixels classified as canopy.** The proportion of canopy pixels dropped between 1984 and 1998, (extreme drought in 1994-95). Then, it increased between 1998 and 2004. Since 2004 the amount of canopy pixels has remained stable, with a small drop between 2013 and 2016 (2012 drought).


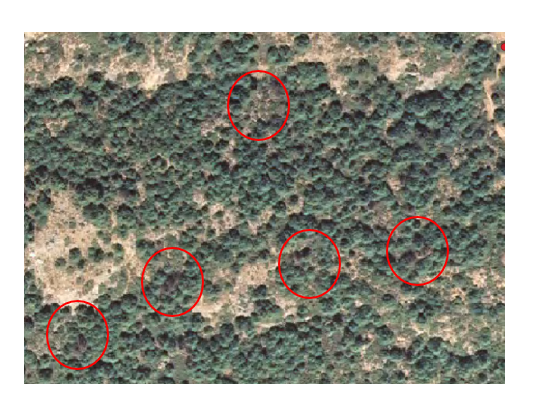


**2019**

**Figure S6. 2007 orthoimage.** Widely spread mortality from the severe 2005 drought can be observed across the whole 2007 orthoimage. Dark pixels are dead trees in the opening gaps process.


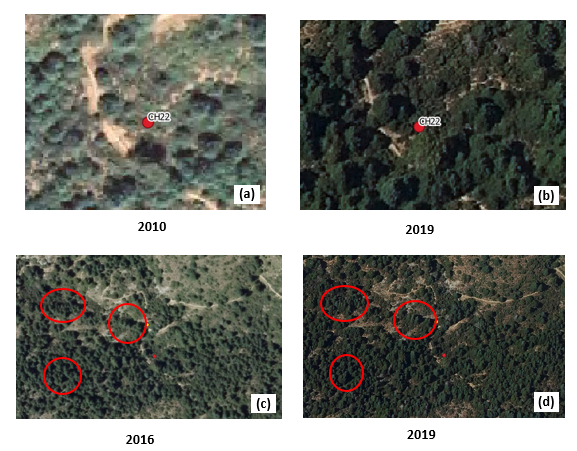


**Figure S7. Two examples of pinsapo tree growth visual evidence.** In (a) and (b) nine years of difference meant bigger crowns and more shrubs in 2019 than in 2010. Meanwhile, in only three years (2016-2019) the rapid crown development between (c) and (d) is evident. All paired images have same scale and, in the pair, (c) and (d) is noticeable this crown development since both orthoimages have the same light angle.
